# On the Question Whether Alkaline Carbonates Exist in the Blood

**Published:** 1846-03

**Authors:** J. Liebig


*On the Question whether Alkaline Carbonates exist in the Blood.* By Baron Liebig.—The food of the carnivora contains only alkaline phosphates; that no alkaline carbonates, therefore, can be found in their blood, scarcely requires any special proof. But the case is altogether with respect to the graminivora, since their food contains a number of alkaline compounds with vegetable acids, which pass into their circulation, and are subsequently separated from the blood through the kidneys, and appear finally in their urine as alkaline carbonates. Now, if these alkaline carbonates exist as constituents of the blood of this class of animals, and if the elimination of carbon in the respiratory process is to be referred to their agency, it is obvious that we must be able to detect and to demonstrate their presence in the blood.

The following experiment was made to determine the question whether alkaline carbonates are present in the blood of graminivorous animals or not, and it must, I think, be deemed conclusive.

A mixture of between four and five pounds of the blood of an ox was boiled with twice its volume of water, and the coagulated mass formed subjected to powerful pressure. The fluid obtained was about the weight of the blood employed in the process; it had a powerfully alkaline reaction. Now, it is evident that this fluid must necessarily contain in solution any alkaline carbonates which existed in the blood.

On evaporation in a retort, (consequently not exposed to contact with the atmosphere,) until it occupied about forty cubic centimetres, it became a thick greenish-brown liquid, of the consistence of syrup, and had still a strong alkaline reaction. I took half this concentrated fluid, and placed it in a graduated tube in contact with carbonic acid gas, and allowed the mixture to stand for twenty-four hours, when I found the fluid had absorbed three times its own volume of the carbonic acid. Now it is evident, that if the capacity of this fluid for absorbing three times its own volume of carbonic acid gas depended on the presence of the carbonate of soda, and, consequently, the formation of bi-carbonate by the addition of the acid, the other half of the liquid obtained from the blood must evolve carbonic acid gas when acted on by a stronger acid, to the extent of about two-thirds that of the gas absorbed by the first half.

But this liquid, when brought into contact with hydrochloric acid in a bell jar over mercury, mixed with the acid without evolving the slightest perceptible trace of carbonic acid.

This experiment confirms the conclusions of Enderlin's analysis of the ashes of the blood of the graminivora—namely, that this blood contains no perceptible amount of alkaline carbonates.—*London Lan.*

